# Hepatitis B and hepatitis C virus infections and associated factors among prisoners in Gondar City, Northwest Ethiopia

**DOI:** 10.1371/journal.pone.0301973

**Published:** 2024-04-16

**Authors:** Kebebe Tadesse, Getnet Ayalew, Yihenew Million, Aschalew Gelaw

**Affiliations:** 1 Department of Medical Microbiology, School of Biomedical and Laboratory Sciences, College of Medicine and Health Science, University of Gondar, Gondar, Ethiopia; 2 Department of Medical Laboratory Sciences, Pawe Health Science College, Pawe, Ethiopia; University of Cincinnati College of Medicine, UNITED STATES

## Abstract

**Background:**

Globally, hepatitis B virus (HBV) and hepatitis C virus (HCV) cause considerable morbidity and mortality from their acute and chronic infections. The transmission of the viruses within the prisons is high due to overcrowding, and other risk behaviors such as drug use, and unsafe sexual practices. This study aimed at determining the prevalence and associated factors of HBV and HCV infections among prisoners in Gondar city, Northwest Ethiopia.

**Methods:**

A cross-sectional study was conducted in the Gondar City Prison Center from May 1, 2022, to July 30, 2022. A total of 299 prison inmates were selected by using a systematic random sampling technique. A semi-structured questionnaire was used to collect data on sociodemographic, clinical, behavioral and prison related factors. Five milliliters of blood sample were collected, and the serum was separated from the whole blood. The serum was tested for HBV surface antigen (HBsAg) and anti-HCV antibody by using an Enzyme-Linked Immunosorbent Assay (ELISA). Data was entered using EpiData version 4.6.0 and exported to SPSS version 20 for analysis. Logistic regression analysis was done to assess the association between the independent variables and HBV and HCV infections. P-values < 0.05 were considered statistically significant.

**Results:**

The overall seroprevalence of HBV or HCV infections was 10.4%. The seroprevalence of HBV and HCV infections was 7.0% and 4.0%, respectively. It has been demonstrated that having several heterosexual partners, sharing sharp materials in prison, having longer imprisonment, and having a body tattoo are significantly associated with HBV infection. The presence of a body tattoo, a history of surgical procedures, and previous imprisonment are associated risk factors for HCV infection.

**Conclusion:**

The prevalence of HBV and HCV were high-intermediate and high, respectively. Therefore, preventative and control initiatives are needed in prisons to decrease the rate of infection and transmission.

## Introduction

Hepatitis is an inflammation of the liver that most frequently occurs during viral infections [1]. Acute and chronic infection due to viral hepatitis are the leading cause of morbidity and mortality worldwide [2]. The most serious are hepatitis B virus (HBV) and hepatitis C virus (HCV) infections [3,4]. Globally, 1.4 million deaths per year are due to viral hepatitis, 96% of which are caused by HBV and HCV infections [4,5]. Both HBV and HCV cause acute and chronic infections, resulting in progressive liver damage, that potentially leads to the development of cirrhosis, hepatocellular carcinoma (HCC), or death [6,7]. Together, HBV and HCV are the most common cause of cirrhosis and HCC. Worldwide, 57% of cirrhosis are due to either HBV (30%) or HCV (27%) and 78% of HCC are due to HBV (53%) or HCV infections (25%) [8,9].

In 2010, viral hepatitis was the tenth leading cause of death, rising to seventh in 2013 [3]. It was the sixth most common cause of death in 2015, accounting for 1.34 million fatalities [2]. According to estimates from the World Health Organization (WHO), in 2019 there were 296 million and 58 million individuals living with HBV and HCV, respectively, with 1.5 million people for each newly developing chronic HBV and HCV infection. An estimated 820 000 and 290 000 people infected with HBV and HCV, respectively, died mostly from cirrhosis and HCC [10–12].

In Africa, 90% of all cases of liver cancer are due to chronic HBV and HCV infections [13]. The estimated prevalence of chronic HBV infection in Africa is 6.1%, with the highest number of infection (78 million) in sub-Saharan Africa [14]. Similarly, there are about 18 million chronic HCV patients in Africa, with sub-Saharan Africa accounting for 20% of the infections [15].

In Ethiopia, HBV and HCV account for more than 60% of chronic liver disease and up to 80% of HCC [16]. The estimated pooled prevalence of HBV and HCV were 6% and 2%, respectively [17,18]. Both HBV and HCV were significant public health problems in Ethiopia. The prevalence of HBV was 4.7% in pregnant women, 4.9% in blood donors, 5% in healthcare workers, and 5% in HIV positive patients [17,19,20]. Prevalence of HCV was estimated to be 1.83% and 0.819% among pregnant women, and blood donors, respectively [21,22]. Despite the high prevalence and availability of antiviral therapies for HBV and HCV infections, diagnosis and treatment of the infections are very low in Ethiopia [23].

The prevalence of HBV and HCV infections among prisoners were reported worldwide. The highest prevalence was reported in the West and Central Africa (23·5%) for HBV and Eastern Europe and Central Asia (20·2%) for HCV [24]. In Ethiopia, studies among prisoners are limited to a few reports [25,26]. According to the 13^th^ version of the world prison population list, more than 10.77 million people are detained in prison around the world in 2021, with 110,000 in Ethiopia being designated as Africa’s third-largest prison population [27]. Prisoners participate in a number of risky behaviors that might result in the spread of HBV and HCV infections, including injecting drug use (IDU), common use of needles for tattoos and skin piercing, sharing items like toothbrushes, and hair clippers [28,29]. Also, prisons increase adverse health conditions through overcrowding, poor infrastructure, lack of adequate infection control practices, and limited or no access to appropriate diagnosis, care, and treatment [30].

The Ethiopian Federal Ministry of Health is currently putting preventive measures in place to stop the spread of viral hepatitis. These include screening of blood and blood products, immunizing infants, and safe injection procedures in medical facilities [31]. In 2007, the Ethiopian Federal Ministry of Health added the HBV vaccine to the standard expanded program on the immunization schedule, targeting children under one year of age [32]. Apart from this, vaccination of HBV is only given to healthcare workers in public health facilities [33]. However, for other high-risk groups such as prisoners no vaccination is given [16]. Currently, the vaccination rate among children and healthcare professionals is 90% and 20%, respectively [33,34]. Prisoners have limited access to diagnosis and preventive measures. Thus, this study was intended to determine the seroprevalence and associated factors of HBV and HCV infections among prisoners in Gondar city, Northwest Ethiopia.

## Methods and materials

### Study design, period and setting

A cross-sectional study was conducted at Gondar City Prison Center from May 1, 2022, to July 30, 2022. Gondar City Prison Center is located in Gondar city, Central Gondar Zone, Amhara National Regional State. Gondar is located in Northwest Ethiopia, 720 km from Addis Ababa and 174 km from Bahir Dar. Gondar city has an estimated population of 395,138 in 2021 [35]. According to the data obtained from the Gondar City Prison Administrative Office, the city has one prison center and more than 3500 (3100 male and 400 female) prisoners were imprisoned during the study period.

### Study population

The study population was prisoners in the Gondar City Prison Center during the study period, who were included in the study by using a systematic sampling technique. Prisoners aged 18 years and older were recruited. Prisoners who stayed in the prison for less than two weeks before data collection and those who had received HBV vaccinations were excluded.

### Sample size determination and sampling technique

The sample size was determined by using single population proportion formula considering 95% confidence level taking the proportion as 6.5% from the previous study in Dessie [25], and a margin of error of 0.03. The formula used to calculate the sample size is as follows: [n = (Z α/2)^2^ p (1-p) / d^2^] = (1.96)^2^ x 0.065(1–0.065)/ (0.03)^2^ = 260. Accordingly, after adding a 15% non-response rate, the final sample size was 299. A systematic random sampling technique was used to recruit the 299 study percipients from the sampling frame formed by the code number of prisoners obtained from the Gondar City Prison Administrative Office. More than 3500 prisoners were incarcerated during the study period. The interval (K) was obtained by dividing the total number of prisoners during the study period by sample size (K = 3500/299 = 11.7 ≈ 12). The first individual was selected by lottery method and the others at regular intervals of 12.

### Data collection

After obtaining signed informed consent, the data was collected by two trained data collectors, using a pre-tested semi-structured questionnaire in a private room at the prison. A face-to-face interview technique was applied to collect data related to socio-demographic, clinical, and prison-related factors and risk behaviors of prisoners. The data about duration of incarceration was obtained from prisoners’ document in the prison.

### Specimen collection and laboratory analysis

Trained laboratory technologists collected five milliliters of venous blood with serum separator tubes (SST) containing a clot activator. The specimen from each study participant was labeled with a unique identification number. The blood specimen was allowed to clot and retract at room temperature for 30 min and centrifuged at 5000 rpm for 5 minutes. The serum was separated from blood cells and stored at -20°C until tested. Finally, the serum was tested for HBsAg using AiD^TM^ HBsAg ELISA and for anti-HCV using AiD^TM^ anti-HCV ELISA^plus^ test kits (Beijing Wantai Biological Pharmacy Enterprise Co., Ltd) at Gondar blood bank services laboratory. A Wantai AiDTM HBsAg ELISA test kit has a sensitivity of 100% and a specificity of 99.92%, and a Wantai AiDTM anti-HCV ELISA^plus^ test kit has a sensitivity of 100% and a specificity of 99.55% were used. All tests were carried out according to the manufacturer’s instruction.

### Data quality control

The questionnaire was first prepared in English and then translated into the Amharic language. The second version of the questionnaire was retranslated into the original one by language experts to evaluate its consistency. Training was given to data collector about data collection procedures and interview techniques. The principal investigators supervised the data collection throughout the study period. To make sure that the questionnaire was appropriate and understandable, it was pretested on 5% of the calculated sample size at the Debark Prison Center before the main study is conducted. Based on the findings of the pre-test, the data collector was reoriented and the questionnaire was modified as necessary.

### Laboratory quality control

All specimens were collected and processed according to the standard operating procedure (SOP). The quality of the specimens was checked to see if they meet the acceptable criteria like clotting and volume. The samples were tested according to the manufacturer’s instruction and all quality issues were maintained by following the SOP for the detection of HBsAg and anti-HCV antibody. The quality of the test result was maintained by using internal quality control of the ELISA and by using known negative and positive samples.

### Data processing and analysis

The data were entered into EpiData version 4.6.0 after being confirmed as complete and were afterward exported to the Statistical Package for Social Sciences (SPSS) version 20 for analysis. The prevalence of HBV and HCV infection was analyzed using frequency analysis. Shapiro-Wilk test was done to check the normality of data. Bivariate logistic regression was done to determine predictors of HBV and HCV infections by calculating the odds ratio (OR). The Hosmer-Lemeshow goodness-of-fit test was used to assess the fitness of the model. The multivariable logistic regression model was then applied to the variables having a p-value of less than or equals to 0.2 in the bivariate, and the adjusted odds ratio (AOR) for those variables was obtained. Variables with p-values less than 0.05 were considered statistically significant.

### Ethical consideration

The ethical approval was obtained from School of Biomedical and Laboratory Sciences, University of Gondar Ethical Review Committee (SBMLS/201). Official permission was obtained from Gondar City Prison Administrative Office. After the explanation of the purpose of the study, all the study participants provided informed written consent. Participation in the study was on a voluntary basis and personal identifiers were not used. All data and specimens collected from study participants were coded, kept private, and only used for the intended research. Prisoners whose blood samples showed positive for HBsAg and/or anti-HCV were referred to prison doctors for further examination and care.

## Results

### Socio-demographic characteristics of prisoners

A total of 299 prisoners participated in the study. The age of the prisoners ranges from 18 to 85 years with the median and interquartile age range (IQR) of 32 (25–43) years. The majority of the study subjects 265 (88.6%) were males, and half of the participants 152 (50.8%) were from rural areas. Most of the participants were married 159 (53.2%). More than one-third, 127 (42.5%) of the prisoners were illiterate. One hundred thirty-seven (45.8%) of the participants were farmers and 80 (26.8%) were self-employed (Table 1).

**Table 1:** Socio-demographic characteristics of prisoners at Gondar City Prison Center, Northwest Ethiopia, 2022.

Variable	Category	Frequency	Percentage
Gender	Male	265	88.6
	female	34	11.4
Age range (in years)	18–24	72	24.1
	25–34	89	29.8
	35–44	70	23.4
	≥45	68	22.7
Marital status	Single	89	29.8
	Married	159	53.2
	Divorced	49	16.4
	Widowed	2	0.7
Residence	Rural	152	50.8
	Urban	147	49.2
Educational status	Illiterate	127	42.5
	Primary school	106	35.5
	Secondary school	50	16.7
	College and above	16	5.4
Occupational status	Student	31	10.4
	Self-employed	80	26.8
	Employed	24	8.0
	Unemployed	12	4.0
	Housewife	15	5.0
	farmer	137	45.8

### Clinical, behavioral, and prison-related factors

A total of 36 (12.0%) participants had a history of previous incarceration. Most of the study participants 189 (63.2%) had been incarcerated for at least more than one year at the time of the data collection. The median (IQR) year of the duration of incarceration was 2 (0.8–3) years with a range of 1 month to 14 years. Among participants, 104(34.8%) had a history of multiple heterosexual partners and 101(33.8%) had experienced body tattoos. Additionally, 86 (28.8%) had a history of sharing sharp materials in prison and 54 (18.1%) had a history of surgical procedures (Table 2).

**Table 2:** Clinical, behavioral, and prison-related factors of prisoners at Gondar City Prison Center, Northwest Ethiopia, 2022.

Variables	Category	Frequency	Percentage
History of previous imprisonment	Yes	36	12.0
	No	263	88.0
Current duration of imprisonment	<1year	110	36.8
	1–3 years	115	38.5
	>3 years	74	24.7
History of dental extraction	Yes	93	31.1
	No	206	68.9
History of blood transfusion	Yes	19	6.4
	No	280	93.6
Surgical procedure	Yes	54	18.1
	No	245	81.9
Sharing sharp materials in prison	Yes	86	28.8
	No	213	71.2
Ever use IDU	Yes	24	8.0
	No	275	92.0
Having body tattoo	Yes	101	33.8
	No	198	66.2
Having body piercing	Yes	66	22.1
	No	233	77.9
Share personal belongings with other	Yes	93	31.1
	No	206	68.9
History of illicit drug use	Yes	39	13.0
	No	260	87.0
Multiple heterosexual partners	Yes	104	34.8
	No	195	65.2
Smoking practice before imprisonment	Yes	46	15.4
	No	253	84.6
Frequent alcohol consumption history	Yes	104	34.8
	No	195	65.2
Contact with the jaundiced patient	Yes	144	48.2
	No	155	51.8
Venous or body piercing for treatment	Yes	84	28.1
	No	215	71.9
History of STI	Yes	52	17.4
	No	247	82.6

IDU: Injecting Drug Use, STI: Sexually Transmitted Infection, Venous or body piercing for treatment: Traditional scarification.

### Prevalence of hepatitis B and hepatitis C viruses

**Fig 1:**
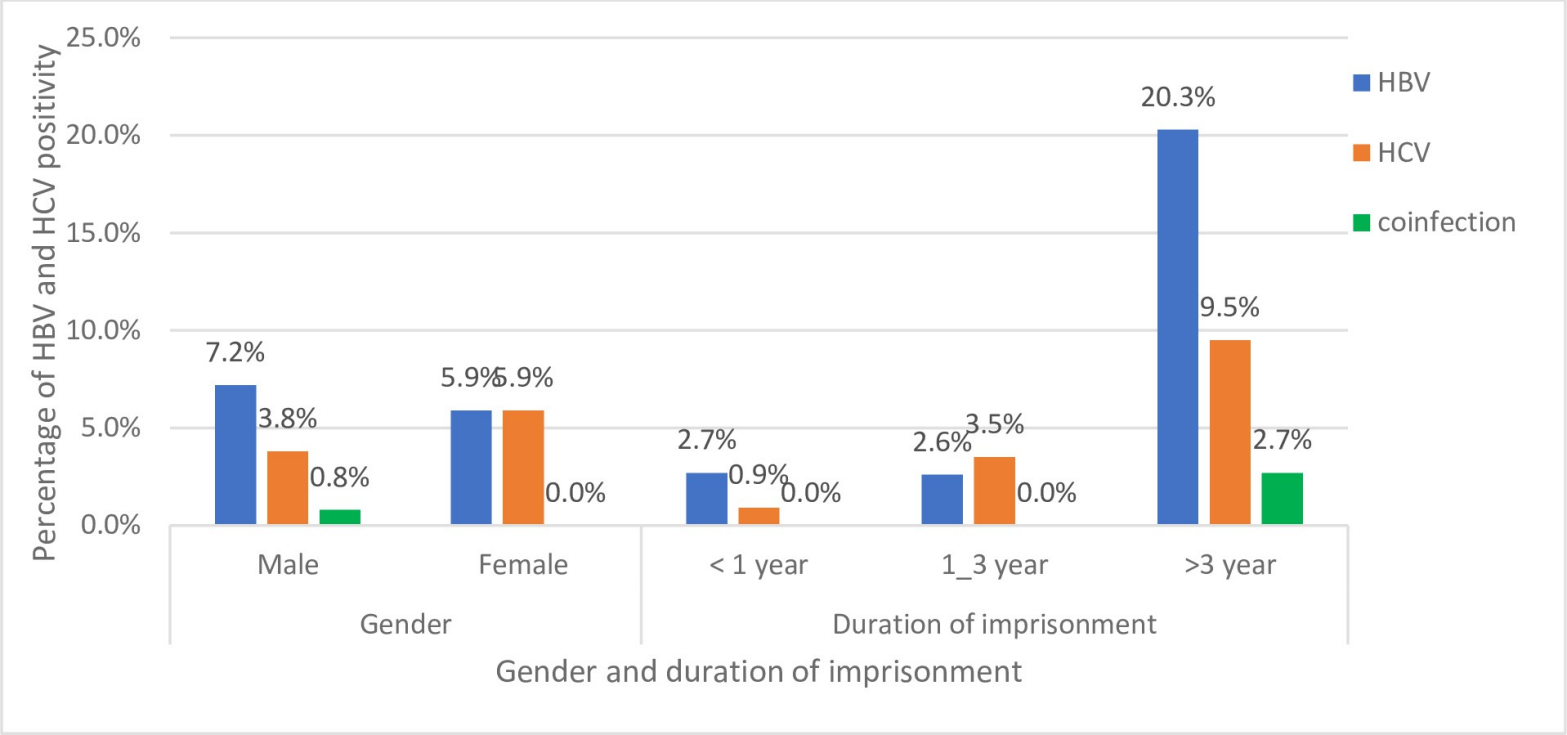
Hepatitis B and hepatitis C virus distribution by gender and duration of imprisonment at Gondar City Prison Center, Northwest Ethiopia, 2022.

The overall seroprevalence of HBV or HCV infections was 10.4% (31/299) (95% CI: 7.4–14.0). The seroprevalence of HBV and HCV was 7.0% (21/299) (95% CI: 4.4–10.5) and 4.0% (12/299) (95% CI: 2.1–6.9), respectively including 0.7% (2/299) coinfections. The prevalence of HBV was higher in males (7.2%) (19/265) than females 5.9% (2/34), while the rate of HCV infection was higher in females (5.9%) (2/34) than males (3.8%) (19/265). The prevalence of both HBV and HCV infections was higher in those prisoners incarcerated for more than three years 20.3% (15/74) and 9.5% (7/74), respectively (Fig 1).

### Factors associated with HBV infection

Determinant variables to HBV infection of prisoners were evaluated by bivariate and multivariate binary logistic regression analysis models. In the binary logistic regression analysis model, none of the sociodemographic variables show a statistically significant association with HBV infection. Besides behavioral and prison-related factors, duration of imprisonment, sharing sharp materials, having body tattoos, multiple heterosexual partners, and a history of STI show statistically significant associations with HBV infection. However, in the multivariable logistic regression analysis model, duration of imprisonment for more than three years (AOR = 6.18; 95%CI: 1.17–22.64), sharing sharp materials in prison (AOR = 4.85; 95%CI: 1.22–19.26), having body tattoo (AOR = 5.29; 95%CI: 1.58–17.71) and having multiple heterosexual partners (AOR = 4.31; 95%CI: 1.10–16.85) have remained statistically associated with HBV infection among prisoners (Table 3).

**Table 3:** Bivariate and multivariate logistic regression analysis of possible risk factors for HBV infection among prisoners in Gondar City Prison Center, Northwest Ethiopia, 2022.

Variables	Category	HBV		COR (95% CI)	P value	AOR (95% CI)		P value
		Positive N (%)	Negative N (%)					
Residence	Rural	14 (66.7)	138 (49.6)	2.03 (0.79–5.18)	0.139		3.11 (0.77–12.51)	0.111
	Urban	7 (33.3)	140 (50.4)	1			1	
Age range (in years)	18–24	4 (19.0)	68 (24.5)	1			1	
	25–34	4 (19.0)	85 (30.6)	0.8 (0.19–3.32)	0.758		0.31 (0.05–1.94)	0.210
	35–44	4 (19.0)	66 (23.7)	1.0 (0.25–4.29)	0.967		0.29 (0.04–1.94)	0.200
	≥45	9 (42.9)	59 (21.2)	2.6 (0.76–8.86)	0.128		0.58 (0.11–3.12)	0.522
History of previous imprisonment	Yes	5 (23.8)	31 (11.2)	2.5 (0.85–7.27)	0.095		2.54 (0.64–10.10)	0.186
	No	16 (76.2)	247 (88.8)	1			1	
Duration of stay in prison	<1year	3 (14.3)	107 (38.5)	1			1	
	1–3 years	3 (14.3)	112 (40.3)	0.96 (0.19–4.84)	0.956		0.79 (0.12–5.29)	0.811
	>3 years	15 (71.4)	59 (21.2)	9.07 (2.52–25.60)	0.001*		6.18 (1.17–22.64)	0.032*
Surgical procedure	Yes	7 (33.3)	47 (16.9)	2.46 (0.94–6.42)	0.066		2.04 (0.56–7.44)	0.279
	No	14 (66.7)	231 (83.1)	1			1	
Sharing sharp materials in prison	Yes	15 (71.4)	71 (25.5)	7.29 (2.72–19.51)	≤0.001*		4.85 (1.22–19.26)	0.025*
	No	6 (28.6)	207 (74.5)	1			1	
Ever use IDU	Yes	4 (19.0)	20 (7.2)	3.04 (0.93–9.89)	0.065		1.65 (0.27–10.00)	0.588
	No	17 (81.0)	258 (92.8)	1			1	
Having body tattoo	Yes	16 (76.2)	85 (30.6)	7.27 (2.58–20.48)	≤0.000*		5.29 (1.58–17.71)	0.007*
	No	5 (23.8)	193 (69.4)	1			1	
Share personal belongings with other	Yes	10 (47.6)	83 (29.9)	2.14 (0.87–5.22)	0.096		0.28 (0.05–1.50)	0.138
	No	11 (52.4)	195 (70.1)	1			1	
History of STI	Yes	8 (38.1)	44 (15.8)	3.27 (1.28–8.36)	0.013*		1.22 (0.29–5.06)	0.780
	No	13 (61.9)	234 (84.2)	1			1	
Contact with jaundiced patient	Yes	13 (61.9)	131 (47.1)	1.8 (0.73–4.54)	0.197		1.53 (0.35–6.68)	0.576
	No	8 (38.1)	147 (52.9)	1			1	
History of multiple heterosexual partners	Yes	15 (71.4)	89 (32.0)	5.3 (1.99–14.14)	0.001*		4.31 (1.10–16.85)	0.036*
	No	6 (28.6)	189 (68.0)	1			1	

AOR: Adjusted odds ratio; COR: Crude odds ratio; HBV: Hepatitis B virus; IDU: Injecting drug use, STI: Sexually transmitted infection

*The observed difference is statistically significant.

### Factors associated with HCV infection

To determine associated factors for HCV infection, both bivariate and multivariate logistic regression analyses were done. Initially, in the bivariate logistic regression analysis; history of previous imprisonment, duration of stay in prison, history of blood transfusion, history of surgical procedure, having body tattoo, and having multiple heterosexual partners showed significant association with the dependent variable. However, there is no bivariate association between any of the socio-demographic features of participants with anti-HCV positivity. The results of multivariate logistic regression showed that HCV exposure had a statistically significant relationship with prisoners with a history of previous imprisonment (AOR = 6.49; 95%CI: 1.34–18.39), surgical procedure (AOR = 5.01; 95%CI: 1.12–22.42), and having body tattoo (AOR = 5.66; 95%CI: 1.18–17.05) (Table 4).

**Table 4:** Bivariate and multivariate logistic regression analysis of possible risk factors for HCV infection among prisoners at Gondar City Prison Center, Northwest Ethiopia, 2022.

Variable	Category	HCV		COR (95% CI)	P value	AOR (95% CI)	P value
		Positive N (%)	Negative N (%)				
History of previous imprisonment	Yes	5 (41.7)	31 (10.8)	5.89 (1.77–19.72)	0.004*	6.49 (1.34–18.39)	0.020*
	No	7 (58.3)	256 (89.2)	1		1	
Current duration of stay in prison	<1year	1 (8.3)	109 (38.0)	1		1	
	1–3 years	4 (33.3)	111 (38.7)	3.93 (0.43–35.71)	0.224	3.18 (0.19–53.58)	0.423
	>3 years	7 (58.3)	67 (23.3)	11.39 (1.37–94.62)	0.024*	12.62 (0.85–187.4)	0.066
History of dental extraction	Yes	6 (50.0)	87 (30.3)	2.29 (0.72–7.33)	0.159	1.63 (0.40–6.65)	0.494
	No	6 (50.0)	200 (69.7)	1		1	
History of blood transfusion	Yes	4 (33.3)	15 (5.2)	9.07 (2.45–33.53)	0.001*	3.35 (0.63–17.72)	0.154
	No	8 (66.7)	272 (94.8)	1		1	
Surgical procedure	Yes	8 (66.7)	46 (16.0)	10.48 (3.03–26.24)	≤0.001*	5.01 (1.12–22.42)	0.035*
	No	4 (33.3)	241 (84.0)	1		1	
Sharing sharp materials in prison	Yes	6 (50.0)	80 (27.9)	2.59 (0.81–8.26)	0.108	1.01 (0.19–5.36)	0.990
	No	6 (50.0)	207 (72.1)	1		1	
Having body tattoo	Yes	9 (75.0)	92 (32.1)	6.35 (1.68–24.04)	0.006*	5.66 (1.18–17.05)	0.030*
	No	3 (25.0)	195 (67.9)	1		1	
share personal belongings with other	Yes	6 (50.0)	87 (30.3)	2.29 (0.72–7.34)	0.159	2.25 (0.43–11.87)	0.339
	No	6 (50.0)	200 (69.7)	1		1	
multiple heterosexual partners	Yes	8 (66.7)	96 (33.4)	3.98 (1.17–13.55)	0.027*	2.06 (0.41–10.45)	0.383
	No	4 (33.3)	191 (66.6)	1		1	
history of STI	Yes	4 (33.3)	48 (16.7)	2.49 (0.72–8.60)	0.149	0.59 (0.10–3.53)	0.562
	No	8 (66.7)	239 (83.3)	1		1	

AOR: Adjusted odds ratio, COR: Crude odds ratio, HCV: Hepatitis C virus, STI: Sexually transmitted infection

*: The observed difference is statistically significant.

## Discussion

Prisons are known to be a high-risk environment for HBV and HCV infections [36]. Indeed, prisoners frequently have chronic HBV and HCV infections [37]. Several factors have been linked to the increased prevalence of HBV and HCV infections among prisoners, such as low socioeconomic status and high-risk behaviors including getting tattoos, using drugs, sharing needles, and engaging in unprotected sexual activity [25,38]. In addition, insufficient living conditions like overcrowding and a lack of early detection and treatment in prisons may contribute to the quicker rate of infection [36,39].

In the current study, a prevalence of 7.0% (95% CI: 4.4–10.5) HBV was found among prisoners in the Gondar City Prison Center. The World Health Organization classified the prevalence of chronic HBV infection as high (≥8%), higher-intermediate (5–7.99%), lower-intermediate (2–4.99%), and low (<2%) [40]. According to this classification, HBV has a higher-intermediate prevalence in the present study. Our estimate is consistent with pooled national prevalence of HBV in Ethiopia 6% and reports from systematic review of HBV among pregnant women 4.7%, blood donors 4.9%, healthcare workers 5%, and HIV positive patients 5% [17,19,20]. In comparison to similar populations, this finding was in agreement with previous literature in Dessie (6.5%), Jimma (5.8%), Woldia (10.4%), and Iran (6.5%) [25,26,41,42]. However, in other studies in Africa and Asia higher prevalence of HBV was obtained among prisoners, 12.5% in West Africa, 16.3% and 13.7% in two Nigeria studies, 12.9% in Cameroon, and 13.6% in Taiwan [37,38,43–45]. These discrepancies may be due to differences in sample size, diagnostic methodology, socioeconomic and cultural factors, the lifestyle of the community, and high-risk behavioral practices such as IDU, homosexuality, and a long stay in the prison which are the primary risk variables that have been discussed in previous studies. Also, it could be explained by differences in the prevalence of infection in communities. In sub-Saharan Africa, Central Africa has the highest prevalence of HBV and followed by West Africa [14]. Additionally, this study did not detect HBV DNA and other serologic markers like anti-HBsAg and anti-HBcAg, which might underestimate the detection rate of the viral infection in this study. On the contrary, studies in Europe had lower HBV prevalence of 0.8%, 1.9%, and 2.6% in Belgium, Sweden, and Turkey, respectively [46–48]. The possible explanation for this great difference might be due to the highest endemic rates of HBV infection in sub-Saharan Africa when compared to European countries [4,46]. The standard of screening procedures, the availability of immunization, and the degree of stakeholder involvement in infection prevention should also be taken into consideration as potential reasons for the low level of prevalence estimates in developed countries [17].

In the present study, the result showed the prevalence of HCV among Gondar city prisoners was 4.0% (95% CI: 2.1–6.9). The World Health Organization classified HCV infection as high, moderate, or low endemic when the prevalence is > 3.5%, 1.5–3.5%, and < 1.5%, respectively [49]. Thus, the finding of the study showed that HCV infection is highly endemic in Gondar city prisoners. This finding is significantly higher than a pooled prevalence in general population (2%), among pregnant women 1.83% and blood donors 0.819% [18,21,22]. Prisoners have a high risk of contracting HCV, thus regular health checks and the detection of infected individuals and identifying associated factors are crucial to preventing the disease and lowering the risk of transmission both within and outside of prisons [50].

Although sparse data exist on HCV prevalence among prisoners in Ethiopia, the results of this study are in accordance with a previous report from Jimma where HCV prevalence among prisoners was estimated at 2.6% [26]. However, the prevalence of HCV infection in this study is higher than the study in Dessie (1.2%) [25]. It might be caused by variations in the prison population like the density of prisoners and risk behaviors among the various prison populations. Furthermore, even though HCV is very common in prisoners and threatens their health status and conditions, there is still no screening program in Ethiopia [51]. The result was comparable with reports from Brazilian and Belgium studies that found a prevalence of 2.4% and 5.0%, respectively [46,52]. However, a low prevalence of HCV infection was observed in this study, than the prevalence of the disease reported in New York (10.1%) [53], California (34.3%) [54], Iran (28%) [55], Taiwan (33.5%) [45], Turkey (17.7%) [48], Sweden (17.0%) [47], and Ireland (22.2%) [56]. The type of prison population examined, based on important risk factors such as IDU, history of imprisonment, and engagement in high-risk sexual behaviors like homosexual activity, may contribute to the disparity in prevalence among studies conducted in various countries [55]. Additionally, the observed variation could be attributed to differences in geographic location, testing methodologies, and sample sizes.

In this study, the socio-demographic characteristics of the participants were not found to be significantly associated with the presence of HBV and HCV infections. However, a study by Kassa et al. suggests that old age is a risk factor for HBV infection [25]. Similarly, studies by Moradi et al. and Puga et al. show that old age was associated with higher HCV infection [52,57]. More than half of our study population was under 35 years might partly explain absence of significant association in this study.

This study demonstrated that the duration of imprisonment was found to be significantly associated with HBV infection. The highest prevalence was observed in those incarcerated for more than three years. This association has also been reported in other previous studies [44,58]. The association between HBV prevalence and prolonged incarceration may be explained by the fact that individuals serving longer sentences are more likely to have prolonged interactions with high-risk populations, thereby increasing their likelihood of encountering risk factors [59].

This study indicates that having a body tattoo is an important risk factor for both HBV and HCV infections. Similar findings of a significant association between having a body tattoo and HBV or HCV infections have been documented by other investigators as well [25,43,56,60]. Tattooing in prisons often involves the use of unsterile equipment, which poses a potential risk for the transmission of blood-borne infections [61]. To minimize the potential harm from these activities, prison officials should provide prisoners with access to harm reduction programs.

This study also found that sharing of sharp materials was significantly associated with HBV infection. Poor living conditions prevailing in prisons include overcrowding often lead to inadequate hygiene practices such as sharing of unsterile razors, needles, and cooking equipment like knives [62]. This practice may be attributed to the various activities carried out in prison which require the use of different sharp materials such as sewing traditional clothes [41]. Hepatitis B virus is highly infectious and can remain stable on environmental surfaces for more than 7 days. Therefore, practices like sharing needles and razors may have contributed to the spread of the virus within this population [43].

Prisoners who engaged in sex with multiple partners had higher odds of HBV infection compared to their counterparts. Similar findings were documented in Dessie and Jimma [25,26]. Increased data suggests that prisoners engage in more sexual activity than the general public, with a greater number of sexual partners and lower condom usage [38].

The present study demonstrated a significantly association between HCV infection and a history of previous imprisonment. Consistently, studies by Khajedaluee et al. and Moradi et al. have reported similar results [39,63]. Multiple incarcerations increase the likelihood of engaging in high-risk behaviors and maylead to drug-related offenses or other confounding factors within the prison, such as violence [64]. Furthermore, the combination of several risk factors and the crowded prison environment may facilitate the transmission of this virus [25].

In this study, prisoners with a history of surgical procedures had five times higher odds of HCV infection compared to those without such history. Similar results were reported by Kazi et al. and Zenebe et al. [65,66]. Failure to adhere to infection control procedures during surgical operations, as well as a gap in the cleaning and sterilizing of materials used in procedures, contribute to increase the transmission of HCV infection [67].

In the present study, IDU was not associated with either HBV or HCV infections. This finding is, however, paradoxical, as several authors have reported an association between IDU and HBV infection [43,68,69]. Similarly, in most studies conducted in the USA [53,54], Europe [56,60], Iran [39,63], and Egypt [70] IDU is identified as the leading risk factor for HCV transmission due to the sharing of needles and drug use equipment. This disparity might be due to low population of IDUs observed in this study sample.

Blood-borne infections such as HCV can spread through blood transfusions. Studies in Jimma and Dessie show that blood transfusion was significantly associated with the transmission of HCV. Contrary, it was not significantly associated with anti-HCV antibody seropositivity in the present study [25,26]. The possible reason for the lack of significant association of HCV infection with blood transfusion might be improved screening of blood before transfusion in this study area.

## Limitations of the study

Limitations of this study include the inability to determine whether the prisoners contracted the virus within or outside of the prison. Reporting bias and social desirability bias may have influenced the vaccination history, as well as clinical and prison-related variables, which were based on self-reporting by the participants. Additionally, due to resource limitations, other HBV markers (anti-HBsAg, anti-HBcAg, and HBV DNA) were not included, thus possibly leading to an underestimation of the actual prevalence of HBV infection. Furthermore, the study may lack representativeness for a wider population due to the inadequate sample size.

## Conclusion

The seroprevalence of HBV and HCV infections among prisoners at the Gondar City Prison Center is relatively high. This high infection rate is associated with a duration of imprisonment, sharing sharp materials in prison, having body tattoos, and having multiple heterosexual partners for HBV infection, whereas, a history of previous imprisonment, history of operation, and having body tattoos are associated with HCV infection. Prison health systems should provide screening, diagnosis, and treatment services for HBV and HCV infections. Also, it is important to set regular primary prevention programs in prisons such as vaccination, and health education to decrease the risk of being infected and prevent the transmission of the disease.

## Supporting information

S1 File (DOCX)

S2 File (DOCX)

## Abbreviations

AOR: Adjusted Odds Ratio
COR: Crud Odds Ratio
DNA: Deoxyribonucleic Acid
ELISA: Enzyme-Linked Immune Sorbent Assay
HBV: Hepatitis B Virus
HBcAg: Hepatitis B virus core Antigen
HBsAg: Hepatitis B virus surface Antigen
HCV: Hepatitis C Virus
HCC: Hepatocellular Carcinoma
HIV: = Human Immunodeficiency Virus
IDU: = Injecting Drug Use
RNA: Ribonucleic Acid
SOP: Standard Operational Procedure
STI: Sexually Transmitted Infection
WHO: World Health Organization
