# Supplementary material for: Hepatitis B and hepatitis C virus infections and associated factors among prisoners in Gondar City, Northwest Ethiopia
Source: PLoS One. 2024 Apr 16;19(4):e0301973. doi: 10.1371/journal.pone.0301973 (PMC11020974; doi:10.1371/journal.pone.0301973)
Supplement: S1 File — (DOCX) [file pone.0301973.s001.docx]

**Annex. Questionnaire English version**

Questionnaires to collect demographic data and associated factors of hepatitis B virus and hepatitis C virus infection among prisoners in Gondar city prison.

Questionnaire number/Code________________

| **Section I: General information on socio-demographic characteristics of the prisoners** | | | |
| --- | --- | --- | --- |
| No | Questions | Coding categories | Remark |
|  | Gender | 1. Male 2. Female |  |
|  | Age | ………. year’s |  |
|  | Marital status | 1. Single 2. Married 3. Divorced 4. Widowed |  |
|  | Residence | 1. Rural 2. Urban |  |
|  | Education status | 1. Illiterate 2. Primary school 3. Secondary school 4. College and above |  |
|  | Occupational status | 1. Student 2. Self-employed 3. Government/private employee 4. Unemployed 5. Housewife 6. Other Specify ……. |  |
| Section 2: Clinical, behavioral, and prison-related factors | | | |
|  | Number of Previous incarcerations | 1. ___________ |  |
|  | Current Duration of imprisonment | 1. ________________ |  |
|  | History of Dental extraction | 1. Yes 2. No |  |
|  | History of blood transfusion | 1. Yes 2. No |  |
|  | History of surgical procedure | 1. Yes 2. No |  |
|  | Sharing sharp materials in prison (e.g., Needles, Razor blades, clippers) | 1. Yes 2. No |  |
|  | Ever use IDU | 1. Yes 2. No |  |
|  | Do you have tattoos on your body? | 1. Yes 2. No |  |
|  | If yes, did you get in prison? | 1. Yes 2. No |  |
|  | Do you have a body piercing? (Ear, Nose) | 1. Yes 2. No |  |
|  | If yes, did you get it in prison? | 1. Yes 2. No |  |
|  | Do you share personal belongings with other inmates e.g., Toothbrushes, Towels? | 1. Yes 2. No |  |
|  | History of illicit drug Use | 1. Yes 2. No |  |
|  | Do you have a history of STI | 1. Yes 2. No |  |
|  | Did you have smoking practice | 1. Yes 2. No |  |
|  | Frequent alcohol consumption history | 1. Yes 2. No |  |
|  | Contact with jaundiced patient | 1. Yes 2. No |  |
|  | Venous or body piercing for treatment | 1. Yes 2. No |  |
|  | Multiple heterosexual partners | 1. Yes 2. No |  |
|  | If yes condom use in extramarital sex | 1. Yes 2. No |  |
|  | Homosexual practice | 1. Yes 2. No |  |
