# Supplementary material for: Hepatitis B and hepatitis C virus infections and associated factors among prisoners in Gondar City, Northwest Ethiopia
Source: PLoS One. 2024 Apr 16;19(4):e0301973. doi: 10.1371/journal.pone.0301973 (PMC11020974; doi:10.1371/journal.pone.0301973)
Supplement: S2 File — (DOCX) [file pone.0301973.s002.docx]

**Annex: Laboratory Standard Operating Procedures**

**Principle of the HBV ELISA test**

For detection of HBsAg, AiDTM HBsAg ELISA uses antibody “sandwich” ELISA method in which, polystyrene microwell strips are pre-coated with monoclonal antibodies specific to HBsAg. A serum sample is added to the microwells. During incubation, the specific immunocomplex formed in case of presence of HBsAg in the sample is captured on the solid phase. Then the second antibody conjugated the enzyme horseradish peroxidase (HRP) directed against a different epitope of HBsAg is added to the wells. During the second incubation step, these HRP-conjugated antibodies will be bound to any HBsAb-HBsAg complexes previously formed during the first incubation, and the unbound HRP-conjugate is then removed by washing. After washing to remove unbound HRP-conjugate, chromogen solutions containing tetramethyl-benzidine (TMB) and urea peroxide are added to the wells. In presence of the antibody-antigen-antibody (HRP) “sandwich” immunocomplex, the colorless chromogens are hydrolyzed by the bound HRP-conjugate to a blue-colored product. The blue color turns yellow after stopping the reaction with sulfuric acid. The amount of color intensity can be measured and it is proportional to the amount of antigen captured in the wells, and to its amount in the sample respectively. Wells containing samples negative for HBsAg remains colorless.

**The test procedure for HBsAg testing**

**Reagent preparation:** allow the reagent to reach room temperature (18-30°C) check the wash buffer concentrate for the presence of salt crystals. If the crystal has formed resolubilizes by warming at 37°C until the crystal dissolve. Dilute the wash buffer by 1:20. Use distilled or deionized water and only clean vessels to dilute the buffer. all other reagents are ready to use as supplied.

**Step 1: preparation**: mark 3 wells as Negative Control, 2 wells as Positive Control, and 1 blank.

**Step 2, Adding diluent**: add 20µl of specimen diluent into each well except the blank

**Step 3, Adding specimen**: Add 100µl of Positive Control, Negative Control, and specimen into their respective wells except for the blank. Mix well by tapping the plate gently. NOTE: Use a new pipette tip after each sampling to avoid cross-contamination.

**Step 4, Incubating:** Cover the plate with the plate cover and incubate at 37°C for 60 minutes.

**Step 5, Adding HRP-conjugate:** at the end of incubation remove and discard the plate cover. Add 50µl HRP-conjugate into each well except the blank and mix by tapping the plate gently.

**Step 6, Incubating**: Cover the plate with the plate cover and incubate at 37°C for 30 minutes

**Step 7, Washing:** at the end of incubation remove and discard the plate cover wash each well 5 times with diluted washing buffer. Each time allow the microwells to soak for 30-60 seconds. After the final washing cycle turns down the plate onto blotting paper or a clean towel and tap it to remove any reminders.

**Step 8, Coloring:** add 50µl of chromogen solution A and 50µl of chromogen solution B into each well including the blank mix gently incubate the plate at 37 for 30 minutes avoiding light the enzyme reaction between the chromogen solution and HRP- conjugate produce a blue color in positive control and HBsAg positive wells.

**Step 9, Stopping reaction**: Add 50µl of stop solution into each well and mix gently. Intensive yellow color develops in positive control and HBsAg positive specimen wells.

**Step 10, Measure the absorbance**: calibrate the plate reader with the blank well and read the absorbance at 450nm. Read the absorbance within 10 minutes after stopping the reaction.

**SPECIMEN COLLECTION, TRANSPORTING AND STORAGE**

1. Specimen Collection: No special preparation required. Collect the specimen in accordance with the normal laboratory practice. Either fresh serum or plasma specimens can be used with this assay. Blood collected by venipuncture should be allowed to clot naturally and completely the serum/plasma must be separated from the clot as early as possible as to avoid hemolysis of the RBC. Care should be taken to ensure that the serum specimens are clear and not ontaminated by microorganisms. Any visible particulate matters in the specimen should be removed by centrifugation at 3000 RPM for 20 minutes at room temperature or by filtration.

2. Plasma specimens collected into EDTA, sodium citrate or heparin may be tested, but highly lipemic, icteric, or hemolytic specimens should not be used as they can give false results in the assay. Do not heat inactivate specimens. This can cause deterioration of the target analyte. Samples with visible microbial contamination should never be used.

3. AiDTM HBsAg ELISA is intended ONLY for testing of individual serum or plasma samples. Do not use the assay for testing of cadaver samples, saliva, urine or other body fluids, or pooled (mixed) blood.

4. Transportation and Storage: Store specimens at 2-8°C. Specimens not required for assaying within 3 days should be stored frozen (-20°C or lower). Multiple freeze-thaw cycles should be avoided. For shipment, samples should be packaged and labeled in accordance with the existing local and international regulations for transportation of clinical samples and ethological agents.

**STORAGE AND STABILITY**

The components of the kit will remain stable through the expiration date indicated on the label and package when stored between 2-8°C; do not freeze. To assure maximum performance of AiDTM HBsAg ELISA, during storage, protect the reagents from contamination with microorganism or chemicals.

**PRECAUTIONS AND SAFETY**

**TO BE USED ONLY BY QUALIFIED PROFESSIONALS**

The ELISA assays are time and temperature sensitive. To avoid incorrect result, strictly follow the test procedure steps and do not modify them.

1. Do not exchange reagents from different lots or use reagents from other commercially available kits. The components of the kit are precisely matched for optimal performance of the tests.

2. Make sure that all reagents are within the validity indicated on the kit box and of the same lot. Never use reagents beyond their expiry date stated on labels or boxes.

3**. CAUTION, CRITICAL STEP:** Allow the reagents and specimens to reach room temperature (18-30°C) before use. Shake reagent gently before use. Return to 2-8°C immediately after use.

4. Use only sufficient volume of sample as indicated in the procedure steps. Failure to do so, may cause low sensitivity of the assay.

5. Do not touch the bottom exterior of the wells; fingerprints or scratches may interfere with the reading. When reading the results, ensure that the plate bottom is dry and there are no air bubbles inside the wells.

6. Never allow the microplate wells to dry after the washing step. Immediately proceed to the next step. Avoid the formation of air bubbles when adding the reagents.

7. Avoid assay steps long time interruptions. Assure same working conditions for all wells.

8. Calibrate the pipette frequently to assure the accuracy of samples/reagents dispensing. Use different disposal pipette tips for each specimen and reagents to avoid cross-contaminations.

9. Assure that the incubation temperature is 37°C inside the incubator.

10. When adding specimens, do not touch the well’s bottom with the pipette tip.

11. When measuring with a plate reader, determine the absorbance at 450nm or at 450/630nm.

12. The enzymatic activity of the HRP-conjugate might be affected from dust and reactive chemical and substances like sodium hypochlorite, acids, alkalis etc. Do not perform the assay in the presence of these substances.

13. If using fully automated equipment, during incubation, do not cover the plates with the plate cover. The tapping out of the remainders inside the plate after washing, can also be omitted.

14. All specimens from human origin should be considered as potentially infectious. Strict adherence to GLP (Good Laboratory Practice) regulations can ensure the personal safety.

15. WARNING: Materials from human origin may have been used in the preparation of the Negative Control of the kit. These materials have been tested with tests kits with accepted performance and found negative for antibodies to HIV 1/2, HCV, TP and HBsAg. However, there is no analytical method that can assure that infectious agents in the specimens or reagents are completely absent. Therefore, handle reagents and specimens with extreme caution as if capable of transmitting infectious diseases. Bovine derived sera have been used for stabilizing of the positive and negative controls. Bovine serum albumin (BSA) and fetal calf sera (FCS) are derived from animals from BSE/TSE free-geographical areas.

16. Never eat, drink, smoke, or apply cosmetics in the assay laboratory. Never pipette solutions by mouth

17. Chemical should be handled and disposed of only in accordance with the current GLP (Good Laboratory Practices) and the local or national regulations.

18. The pipette tips, vials, strips and specimen containers should be collected and autoclaved for not less than 2 hours at 121°C or treated with 10% sodium hypochlorite for 30 minutes to decontaminate before any further steps of disposal. Solutions containing sodium hypochlorite should NEVER be autoclaved. Materials Safety Data Sheet (MSDS) available upon request.

19. Some reagents may cause toxicity, irritation, burns or have carcinogenic effect as raw materials. Contact with the skin and the mucosa should be avoided but not limited to the following reagents: Stop solution, the Chromogens, and the Wash buffer.

20. The Stop solution 0.5M H2SO4 is an acid. Use it with appropriate care. Wipe up spills immediately and wash with water if stop solution comes into contact with the skin or eyes.

21. ProClinTM 300 0.1% used as preservative, can cause irritation of the skin. Wipe up spills immediately or wash with water if solution comes into contact with the skin or eyes.

**INDICATIONS OF INSTABILITY DETERIORATION OF**

**THE REAGENT**: Values of the Positive or Negative controls, which are out of the indicated quality control range, are indicators of possible deterioration of the reagents and/or operator or equipment errors. In such case, the results should be considered as invalid and the samples must be retested. In case of constant erroneous results and proven deterioration or instability of the reagents, immediately substitute the reagents with new one or contact Wantai technical support for further assistance.

**INSTRUCTIONS FOR WASHING**

1. A good washing procedure is essential to obtain correct and precise analytical data.

2. It is recommended to use a good quality ELISA microplate washer, maintained at the best level of washing performances. In general, no less than 5 automatic washing cycles of 350-400μl/well are sufficient to avoid false positive reactions and high background.

3. To avoid cross-contaminations of the plate with specimen or HRP-conjugate, after incubation, do not discard the content of the wells but allow the plate washer to aspirate it automatically.

4. Assure that the microplate washer liquid dispensing channels are not blocked or contaminated and sufficient volume of Wash buffer is dispensed each time into the wells.

5. In case of manual washing, we suggest carrying out 5 washing cycles, dispensing 350-400μl/well and aspirating the liquid for 5 times. If poor results (high background) are observed, increase the wash cycles or soaking time per well.

6. In any case, the liquid aspirated out of the strips should be treated with a sodium hypochlorite solution at a final concentration of 2.5% for 24 hours, before they are wasted in an appropriate way.

7. The concentrated Wash buffer should be diluted 1:20 before use. If less than a whole plate is used, prepare the proportional volume of solution.

**QUALITY CONTROL AND CALCULATION OF THE RESULTS**

Each microplate should be considered separately when calculating and interpreting the results of the assay, regardless of the number of plates concurrently processed. The results are calculated by relating each specimen absorbance (A) value to the Cut-off value (C.O.) of the plate. If the Cut-off reading is based on a single filter plate reader, the results should be calculated by subtracting the Blank well A value from the print report values of specimens and controls. In case the reading is based on dual filter plate reader, do not subtract the Blank well A value from the print report values of specimens and controls.

Calculation of the Cut-off value (C.O.) = Nc + 0.06 (Nc = the mean absorbance value for three negative controls).

Quality control (assay validation): The test results are valid if the Quality Control criteria are fulfilled. It is recommended that each laboratory must establish appropriate quality control system with quality control material similar to, or identical with the sample being analyzed.

- The A value of the Blank well, which contains only Chromogen and Stop solution, is < 0.080 at 450 nm.
- The A values of the Positive control must be ≥ 0.800 at 450/630nm or at 450nm after blanking.
- The A values of the Negative control must be < 0.100 at 450/630nm or at 450nm after blanking.

If one of the Negative control A values does not meet the Quality Control criteria, it should be discarded and the mean value calculated again using the remaining two values. If more than one Negative control A values do not meet the Quality Control Range specifications, the test is invalid and must be repeated.

**1. Quality Control**

Blank well A value: A1= 0.025 at 450nm (Note: blanking is required only when reading with single filter at 450nm)

Well No.: B1, C1, D1

Negative control A values after blanking: 0.020, 0.012, 0.016

Well No.: E1 F1

Positive control A values after blanking: 2.421, 2.369

All control values are within the stated quality control range

2. Calculation of Nc: = (0.020+0.012+0.016)/3 = 0.016

3. Calculation of the Cut-off: (C.O.) = 0.016 +0.06 = 0.076

**INTERPRETATIONS OF THE RESULTS**

Negative Results (A / C.O. < 1): Specimens giving absorbance less than the Cut-off value are negative for this assay, which indicates that no hepatitis B virus surface antigen has been detected with AiDTM HBsAg ELISA, therefore the sample is probably not infected with HBV and the blood unit does not contain hepatitis B virus surface antigen.

Positive Results (A / C.O. ≥ 1): Specimens giving an absorbance equal to or greater than the Cut-off value are considered initially reactive, which indicates that hepatitis B virus surface antigen has probably been detected using AiDTM HBsAg ELISA. All initially reactive specimens should be retested in duplicates using AiDTM HBsAg ELISA before the final assay results are interpreted. Repeatedly reactive specimens can be considered positive for hepatitis B virus surface antigen with AiDTM HBsAg ELISA.

Borderline (A / C.O. = 0.9-1.1): Specimens with absorbance to Cut-off ratio between 0.9 and 1.1 are considered borderline and retesting of these specimens in duplicates is required to confirm the initial results. Follow-up, confirmation and supplementary testing of any positive specimen with other analytical system (e.g. PCR) is required. Conclusions should not be established based on a single test result. It should integrate other laboratory data and findings.

If, after retesting of the initially reactive samples, both wells are negative results (A/C.O.<0.9), these samples should be considered as non-repeatable positive (or false positive) and recorded as negative. As with many very sensitive ELISA assays, false positive results can occur due to the several reasons, most of which are connected with, but not limited to, inadequate washing.

If after retesting in duplicates, one or both wells are positive results, the final result from this ELISA test should be recorded as repeatedly reactive. Repeatedly reactive specimens could be considered positive for hepatitis B virus surface antigen and therefore the sample is probably infected with HBV.

After retesting in duplicates, samples with values close to the Cut-off value should be interpreted with caution and considered as "borderline" zone sample, or uninterpretable for the time of testing.

**Principle of the anti-HCV ELISA test**

This kit is a two-step incubation enzyme immunoassay, which uses polystyrene microwell strips pre-coated with recombinant HCV antigens expressed in E. coli (recombinant Core and NS3/4/5). A serum sample is added together with biotin-conjugated HCV antigens. During the first incubation step, the specific HCV antibodies, if present, will be captured inside the wells as a double antigen “sandwich” complex comprising of the coated, and the biotin-conjugated HCV antigens. The microwells are then washed to remove unbound serum proteins. During the second incubation step, the captured HCV antibodies are detected by adding of HRP- Conjugate. The microwells are then washed to remove unbound conjugate, and Chromogen solutions are added to the wells. In wells positive for HCV antibodies, the colorless Chromogens are hydrolyzed by the bound HRP conjugate to a blue colored product. The blue color turns yellow after stopping the reaction with sulfuric acid. The amount of color intensity can be measured and is proportional to the amount of antibodies captured in the wells, and to the sample respectively. Wells containing samples negative for anti HCV remain colorless.

**The test procedure for the anti-HCV test**

**Reagent preparation:** allow the reagent to reach room temperature (18-30°C) check the wash buffer concentrate for the presence of salt crystals. If crystals have formed resolubilize by warming at 37°C until the crystal dissolve. Dilute the wash buffer by 1:20. Use distilled or deionized water and only clean vessels to dilute the buffer. all other reagents are ready to use as supplied.

**Step 1: preparation**: mark 3 wells as Negative Control, 2 wells as Positive Control, and 1 blank.

**Step 2, Adding** **BIOTIN-conjugate reagent**: add 50µl of BIOTIN-conjugate reagent into each well except the blank

**Step 3, Adding specimen**: Add 50µl of Positive Control, Negative Control, and specimen into their respective wells except for the blank. Mix well by tapping the plate gently. NOTE: Use a new pipette tip after each sampling to avoid cross-contamination.

**Step 4, Incubating:** Cover the plate with the plate cover and incubate at 37°C for 60 minutes.

**Step 5, Washing:** at the end of incubation remove and discard the plate cover wash each well 5 times with diluted washing buffer. Each time allow the microwells to soak for 30-60 seconds. After the final washing cycle turns down the plate onto blotting paper or a clean towel and tap it to remove any reminders.

**Step 6, Adding HRP-conjugate:** Add 100 µl of HRP-conjugate into each well except the blank.

**Step 7, Incubating**: Cover the plate with the plate cover and incubate at 37°C for 30 minutes

**Step 8, Washing:** at the end of incubation remove and discard the plate cover wash each well 5 times with diluted washing buffer. Each time allow the microwells to soak for 30-60 seconds. After the final washing cycle turns down the plate onto blotting paper or a clean towel and tap it to remove any reminders.

**Step 9, Coloring:** add 50µl of chromogen solution A and 50µl of chromogen solution B into each well including the blank, mix gently incubate the plate at 37 for 30 minutes avoiding light. The enzymatic reaction between the chromogen solution and HRP- conjugate produces blue color in positive control and HBsAg positive wells.

**Step 10, Stopping reaction**: Add 50µl of stop solution into each well and mix gently. Intensive yellow color develops in positive control and HBsAg positive specimen wells.

**Step11, Measure the absorbance**: calibrate the plate reader with the blank well and read the absorbance at 450nm. Calculate the cut-off value and evaluate the results. Read the absorbance within 10 minutes after stopping the reaction

**SPECIMEN COLLECTION, TRANSPORTING AND STORAGE**

1. Specimen Collection: No special preparation required. Collect the specimen in accordance with the normal laboratory practice. Either fresh serum or plasma specimens can be used with this assay. Blood collected by venipuncture should be allowed to clot naturally and completely the serum/plasma must be separated from the clot as early as possible as to avoid hemolysis of the RBC. Care should be taken to ensure that the serum specimens are clear and not ontaminated by microorganisms. Any visible particulate matters in the specimen should be removed by centrifugation at 3000 RPM for 20 minutes at room temperature or by filtration.

2. Plasma specimens collected into EDTA, sodium citrate or heparin may be tested, but highly lipemic, icteric, or hemolytic specimens should not be used as they can give false results in the assay. Do not heat inactivate specimens. This can cause deterioration of the target analyte. Samples with visible microbial contamination should never be used.

3. AiD^TM^ anti-HCV ELISA ^plus^ is intended ONLY for testing of individual serum or plasma samples. Do not use the assay for testing of cadaver samples, saliva, urine or other body fluids, or pooled (mixed) blood.

4. Transportation and Storage: Store specimens at 2-8°C. Specimens not required for assaying within 3 days should be stored frozen (-20°C or lower). Multiple freeze-thaw cycles should be avoided. For shipment, samples should be packaged and labeled in accordance with the existing local and international regulations for transportation of clinical samples and ethological agents.

**STORAGE AND STABILITY**

The components of the kit will remain stable through the expiration date indicated on the label and package when stored between 2-8°C; do not freeze. To assure maximum performance of AiD^TM^ anti-HCV ELISA ^plus^, during storage, protect the reagents from contamination with microorganism or chemicals.

**PRECAUTIONS AND SAFETY**

**TO BE USED ONLY BY QUALIFIED PROFESSIONALS**

The ELISA assays are time and temperature sensitive. To avoid incorrect result, strictly follow the test procedure steps and do not modify them.

1. Do not exchange reagents from different lots or use reagents from other commercially available kits. The components of the kit are precisely matched for optimal performance of the tests.

2. Make sure that all reagents are within the validity indicated on the kit box and of the same lot. Never use reagents beyond their expiry date stated on labels or boxes.

3**. CAUTION, CRITICAL STEP:** Allow the reagents and specimens to reach room temperature (18-30°C) before use. Shake reagent gently before use. Return to 2-8°C immediately after use.

4. Use only sufficient volume of sample as indicated in the procedure steps. Failure to do so, may cause low sensitivity of the assay.

5. Do not touch the bottom exterior of the wells; fingerprints or scratches may interfere with the reading. When reading the results, ensure that the plate bottom is dry and there are no air bubbles inside the wells.

6. Never allow the microplate wells to dry after the washing step. Immediately proceed to the next step. Avoid the formation of air bubbles when adding the reagents.

7. Avoid assay steps long time interruptions. Assure same working conditions for all wells.

8. Calibrate the pipette frequently to assure the accuracy of samples/reagents dispensing. Use different disposal pipette tips for each specimen and reagents to avoid cross-contaminations.

9. Assure that the incubation temperature is 37°C inside the incubator.

10. When adding specimens, do not touch the well’s bottom with the pipette tip.

11. When measuring with a plate reader, determine the absorbance at 450nm or at 450/630nm.

12. The enzymatic activity of the HRP-conjugate might be affected from dust and reactive chemical and substances like sodium hypochlorite, acids, alkalis etc. Do not perform the assay in the presence of these substances.

13. If using fully automated equipment, during incubation, do not cover the plates with the plate cover. The tapping out of the remainders inside the plate after washing, can also be omitted.

14. All specimens from human origin should be considered as potentially infectious. Strict adherence to GLP (Good Laboratory Practice) regulations can ensure the personal safety.

15. WARNING: Materials from human origin may have been used in the preparation of the Negative Control of the kit. These materials have been tested with tests kits with accepted performance and found negative for antibodies to HIV 1/2, HCV, TP and HBsAg. However, there is no analytical method that can assure that infectious agents in the specimens or reagents are completely absent. Therefore, handle reagents and specimens with extreme caution as if capable of transmitting infectious diseases. Bovine derived sera have been used for stabilizing of the positive and negative controls. Bovine serum albumin (BSA) and fetal calf sera (FCS) are derived from animals from BSE/TSE free-geographical areas.

16. Never eat, drink, smoke, or apply cosmetics in the assay laboratory. Never pipette solutions by mouth

17. Chemical should be handled and disposed of only in accordance with the current GLP (Good Laboratory Practices) and the local or national regulations.

18. The pipette tips, vials, strips and specimen containers should be collected and autoclaved for not less than 2 hours at 121°C or treated with 10% sodium hypochlorite for 30 minutes to decontaminate before any further steps of disposal. Solutions containing sodium hypochlorite should NEVER be autoclaved. Materials Safety Data Sheet (MSDS) available upon request.

19. Some reagents may cause toxicity, irritation, burns or have carcinogenic effect as raw materials. Contact with the skin and the mucosa should be avoided but not limited to the following reagents: Stop solution, the Chromogens, and the Wash buffer.

20. The Stop solution 0.5M H2SO4 is an acid. Use it with appropriate care. Wipe up spills immediately and wash with water if stop solution comes into contact with the skin or eyes.

21. ProClin^TM^ 300 0.1% used as preservative, can cause irritation of the skin. Wipe up spills immediately or wash with water if solution comes into contact with the skin or eyes.

**INDICATIONS OF INSTABILITY DETERIORATION OF**

**THE REAGENT**: Values of the Positive or Negative controls, which are out of the indicated quality control range, are indicators of possible deterioration of the reagents and/or operator or equipment errors. In such case, the results should be considered as invalid and the samples must be retested. In case of constant erroneous results and proven deterioration or instability of the reagents, immediately substitute the reagents with new one or contact wantai technical support for further assistance.

**INSTRUCTIONS FOR WASHING**

1. A good washing procedure is essential to obtain correct and precise analytical data.

2. It is recommended to use a good quality ELISA microplate washer, maintained at the best level of washing performances. In general, no less than 5 automatic washing cycles of 350-400μl/well are sufficient to avoid false positive reactions and high background.

3. To avoid cross-contaminations of the plate with specimen or HRP-conjugate, after incubation, do not discard the content of the wells but allow the plate washer to aspirate it automatically.

4. Assure that the microplate washer liquid dispensing channels are not blocked or contaminated and sufficient volume of Wash buffer is dispensed each time into the wells.

5. In case of manual washing, we suggest carrying out 5 washing cycles, dispensing 350-400μl/well and aspirating the liquid for 5 times. If poor results (high background) are observed, increase the wash cycles or soaking time per well.

6. In any case, the liquid aspirated out of the strips should be treated with a sodium hypochlorite solution at a final concentration of 2.5% for 24 hours, before they are wasted in an appropriate way.

7. The concentrated Wash buffer should be diluted 1:20 before use. If less than a whole plate is used, prepare the proportional volume of solution.

**QUALITY CONTROL AND CALCULATION OF THE RESULTS**

Each microplate should be considered separately when calculating and interpreting the results of the assay, regardless of the number of plates concurrently processed. The results are calculated by relating each specimen absorbance (A) value to the Cut-off value (C.O.) of the plate. If the Cut-off reading is based on a single filter plate reader, the results should be calculated by subtracting the Blank well A value from the print report values of specimens and controls. In case the reading is based on dual filter plate reader, do not subtract the Blank well A value from the print report values of specimens and controls.

Calculation of the Cut-off value (C.O.) = Nc + 0.12 (Nc = the mean absorbance value for three negative controls).

**Quality control (assay validation):** The test results are valid if the Quality Control criteria are fulfilled. It is recommended that each laboratory must establish appropriate quality control system with quality control material similar to, or identical with the sample being analyzed.

- The A value of the Blank well, which contains only Chromogen and Stop solution, is < 0.080 at 450 nm.
- The A values of the Positive control must be ≥ 0.800 at 450/600-650nm or at 450nm after blanking.
- The A values of the Negative control must be < 0.100 at 450/600-650nm or at 450nm after blanking.

If one of the Negative control A values does not meet the Quality Control criteria, it should be discarded and the mean value calculated again using the remaining two values. If more than one Negative control A values do not meet the Quality Control Range specifications, the test is invalid and must be repeated.

**1. Quality Control**

Blank well A value: A1= 0.025 at 450nm (Note: blanking is required only when reading with single filter at 450nm)

Well No.: B1, C1, D1

Negative control A values after blanking: 0.020, 0.012, 0.016

Well No.: E1 F1

Positive control A values after blanking: 2.421, 2.369

All control values are within the stated quality control range

**2. Calculation of Nc**: = (0.020+0.012+0.016)/3 = 0.016

**3. Calculation of the Cut-off:** (C.O.) = 0.016 +0.12 = 0.136

**INTERPRETATIONS OF THE RESULTS**

**Negative Results** (A / C.O. < 1): Specimens giving absorbance less than the Cut-off value are negative for this assay, which indicates that no hepatitis C virus antibodies have been detected with AiD^TM^ anti-HCV ELISA ^plus^, therefore the sample is probably not infected with HCV and the blood unit does not contain hepatitis C virus antibodies.

**Positive Results** (A / C.O. ≥ 1): Specimens giving an absorbance equal to or greater than the Cut-off value are considered initially reactive, which indicates that hepatitis C virus antibodies have probably been detected using AiD^TM^ anti-HCV ELISA ^plus^. All initially reactive specimens should be retested in duplicates using AiD^TM^ anti-HCV ELISA ^plus^ before the final assay results are interpreted. Repeatedly reactive specimens can be considered positive for hepatitis C virus antibodies with AiD^TM^ anti-HCV ELISA ^plus^.

Borderline (A / C.O. = 0.9-1.1): Specimens with absorbance to Cut-off ratio between 0.9 and 1.1 are considered borderline and retesting of these specimens in duplicates is required to confirm the initial results. Follow-up, confirmation and supplementary testing of any positive specimen with other analytical system (e.g. PCR) is required. Conclusions should not be established based on a single test result. It should integrate other laboratory data and findings.

If, after retesting of the initially reactive samples, both wells are negative results (A/C.O.<0.9), these samples should be considered as non-repeatable positive (or false positive) and recorded as negative. As with many very sensitive ELISA assays, false positive results can occur due to the several reasons, most of which are connected with, but not limited to, inadequate washing.

If after retesting in duplicates, one or both wells are positive results, the final result from this ELISA test should be recorded as repeatedly reactive. Repeatedly reactive specimens could be considered positive for hepatitis C virus antibodies and therefore the sample is probably infected with HCV.

After retesting in duplicates, samples with values close to the Cut-off value should be interpreted with caution and considered as "borderline" zone sample, or uninterpretable for the time of testing.
